# The role of ^18^F-FDG PET CT in common gynaecological malignancies

**DOI:** 10.1259/bjr.20170283

**Published:** 2017-10-20

**Authors:** Priya Narayanan, Anju Sahdev

**Affiliations:** 1 Department of Imaging, University College Hospital NHS Trust, London, UK; 2 Department of Imaging, St Bartholomew’s Hospital, Barts Health NHS Trust, London, UK

## Abstract

[fluorine-18]-fluoro-2-deoxy-D-glucose positron emission tomography (^18^F-FDG PET CT) has increasing clinical applications supplementing conventional TVUS, CT and MRI imaging in assessing ovarian, cervical and endometrial cancer. The published literature on the applications of ^18^F-FDG PET CT shows its use can have significant impact on patient management by improving staging of the cancers, influencing patient selection for treatment and in detecting early recurrent disease. However, the increasing clinical use of PET CT does not always align with the guidelines, recommendations or expert opinion in the use of PET CT. This article summarizes the existing evidence base for the established clinical applications and the emerging roles for ^18^F-FDG PET CT in the common gynaecological malignancies.

## Introduction

For imaging gynaecological malignancies, ultrasound, CT and MRI are the established imaging techniques. More recently, positron emission tomography CT (PET CT) has increasing clinical application to supplement conventional imaging in assessing ovarian, cervical and endometrial cancer. [fluorine-18]-fluoro-2-deoxy-D-glucose (^18^F-FDG PET CT) provides key information in staging and therapy monitoring of many tumours, such as lung carcinoma, colorectal cancer, lymphoma, gynaecological cancers, melanoma and many others. The commonly used current recommendations for the use of ^18^F-FDG PET CT is summarized in (Table 1) . The published literature on the applications of ^18^F-FDG PET CT in gynaecological malignancies is still maturing and recent literature has shown its use can have significant impact on patient management. Its use can improve staging, influence patient selection for therapies and detect early recurrent disease. However, the increasing clinical use does not always align with the guidelines, recommendations or expert opinion in the use of PET CT. This article will summarize the emerging clinical use of ^18^F-FDG PET CT in ovarian, endometrial and cervical cancer discussing the supporting evidence for its use.

**Table 1.** Current indications for the use of PET CT in the gynaecological malignancies, based on the collaborative document: evidence-based indications for the use of PET CT in the UK 2016, SIGN 2008, American NCCN guidelines

	**RCR collaborative use of P ET CT UK 2016**	**SIGN guidelines**	**American NCCN guidelines endometrial cancer:version 2017**
Endometrial cancer	No role in primary staging. Suspected recurrence with equivocal cross-sectional imaging. Patients being considered for exenterative surgery	No specific guidelines for endometrial cancer. Related guidelines for post menopausal bleeding does not recommend PET CT at initial diagnosis or staging of endometrial cancer	PET CT at initial staging or at suspected recurrence if metastatic disease is suspected in select patients
	**RCR collaborative use of PET CT UK 2016**	**SIGN guidelines ovarian cancer 2013**	**American NCCN guidelines ovarian cancer**
Ovarian cancer	No role in primary staging. Rising CA125 with negative or equivocal cross-sectional imaging	Not routinely recommended in the diagnosis, staging, surveillance or at recurrence of epithelial ovarian cancer	PET/CT not routine for characterizing adnexal lesions but may be considered if results will alter management. Not routinely recommended for initial staging, surveillance following complete response. Serially rising CA-125 with or without previous chemotherapy. Clinical relapse with or without previous chemotherapy
	**RCR collaborative use of PET CT UK 2016**	**SIGN guidelines ovarian cancer 2013**	**American NCCN guidelines c ervical cancer:version 2016–17**
Cervical cancer	Suspected recurrence with equivocal cross-sectional imaging. Patients being considered for exenterative surgery. Response assessment of locally advanced cervical cancer following chemoradiotherapy	Stage IB or IIA (tumour > 2 cm) being considered for surgery to determine the extent of potential metastatic involvement. Patients with inoperable disease, considered potentially curable with chemoradiotherapy. This group is more likely to have nodal or metastatic disease than those suitable for surgery. 9 months surveillance PET CT is recommended in women who have had chemoradiotherapy. All patients in whom recurrent or persistent disease has been detected on MRI or CT and salvage therapy (either pelvic exenteration or radiotherapy) are being considered	At initial presentation for confirmed or incidentally detected (at hysterectomy) cervical cancer, Consider PET/CT in FIGO Stage IB2-IV to evaluate metastatic disease and to help define nodal volume of coverage for radiotherapy. For surveillance and follow-up: For patients on fertility sparing treatment, consider PET CT only if metastases are suspected. In patients with FIGO Stage IB2 with high risk factors and patients with FIGO stage II-IV who have received post-operative adjuvant radiation or chemoradiation, 3–6 months after completion of treatment. Symptomatic suspicion of local recurrence

FIGO, International Federation of Gynecology and Obstetrics; NCCN, National Comprehensive Cancer Network; PET CT, positron emission tomography; RCR, Royal College of Radiologists; SIGN, Scottish Intercollegiate Guidelines Network; TVUS, Transvaginal ultrasound.

## ^18^F-FDG PET CT -Pitfalls and limitations in gynaecological malignancies

The advantage of ^18^F-FDG PET CT over conventional cross-sectional imaging is its ability to combine anatomical and metabolic information.^18^F-FDG PET-CT has seen increasing clinical popularity as the functional PET component improves specificity and sensitivity, while CT provides anatomical specificity in tumour imaging. The specificity of ^18^F-FDG PET CT is lowered by concurrent infective and inflammatory processes, which are seen at presentation of malignancy or manifest secondary to treatment effects. In addition, there are specific recognized pitfalls related to gynaecological imaging, which the reporting radiologist should be familiar with in order to avoid errors in interpretation. Many of these changes are secondary to physiological variations in the normal menstrual cycle and concurrent infective or post treatment changes. Pelvic inflammatory changes are frequently likely to co-exist in patients presenting with ovarian and cervical cancer. These pitfalls and limitations are summarized in Table 2.

**Table 2:** Limitations of ^18^F-FDG PET CT in gynaecological malignancies

Poor spatial resolution and respiratory motion artefact limits identification of sub diaphragmatic peritoneal disease, sub centimetre peritoneal deposits and lung nodules		
Inflammatory and infective pelvic pathology may demonstrate ^18^F-FDG PET CT uptake thereby causing false positive results		
Physiological cyclical uptake of ^18^F-FDG uptake seen in normal endometrium during the ovulatory and menstrual phases. Physiological uptake of ^18^F-FDG uptake in normal ovaries in the follicular and luteal phases		
^18^F-FDG uptake by benign leiomyomas		
Physiological excretion of FDG accumulating in the bladder may mask small pelvic masses, parametrial disease or parametrial nodes in endometrial and cervical cancers		
Focal ureteric excretion may be misinterpreted as metabolically active metastatic lymph nodes		
Metallic implants/devices can result in attenuation correction artefacts on PET CT masking small volume peritoneal disease		
Primary mucinous ovarian tumours, nodal and peritoneal metastases from mucinous carcinomas and necrotic lymph nodes may have no or very low FDG uptake and may be missed		

## Role of ^18^F-FDG PET CT In ovarian cancer

Ovarian cancer is the seventh most common cancer for females, with nearly 239,000 new cases diagnosed in 2012 worldwide.^1^ The use of ^18^F-FDG PET CT has been advocated for characterizing adnexal masses, staging ovarian cancer and detecting recurrent disease in the context of rising CA125.

### Characterizing adnexal masses

**Figure 1.**
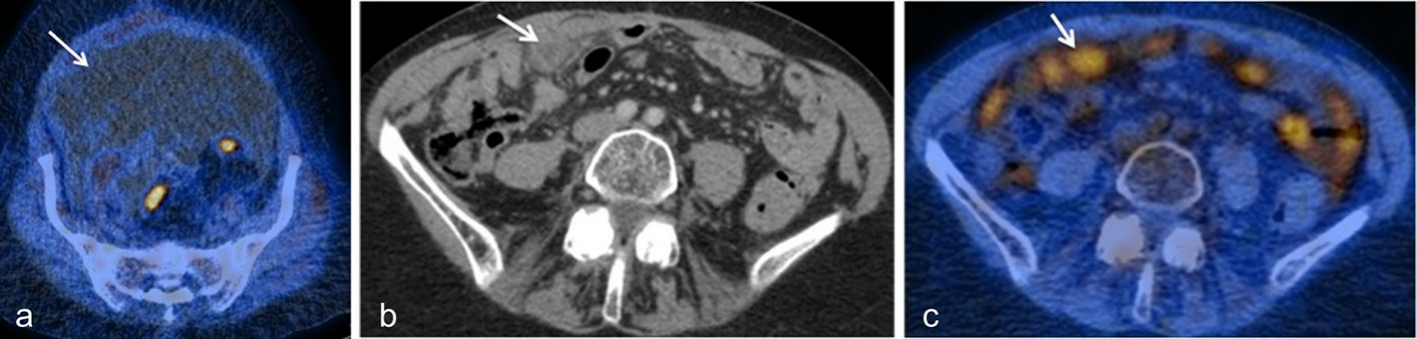
A 67-year-old female with endometriod carcinoma of the ovary. (a) The primary disease in the pelvis is poorly FDG avid (arrow). (b, c) A cystic deposit in the serosa, equally poorly FDG avid and therefore masked by bowel, better demonstrated on the CECT. CECT, contrast enhanced CT; FDG, fluoro-2-deoxy-D-glucose.

Ultrasound remains the first line imaging modality for detection and characterization of ovarian masses with MRI used to evaluate indeterminate lesions.^2^ Early studies demonstrated the performance of ^18^F-FDG PET CT was inferior to MRI in characterizing adnexal masses. Rieber et al found ^18^F-FDG PET CT in characterizing a pelvic mass deemed suspicious on ultrasound had a sensitivity of 58% and a specificity of 78%.^3^ Hubner et al had similar results with a positive predictive value of PET CT for ovarian cancer of 86% and the negative predictive value of 76%^4^ (Figure 1). As expected, false positive results were obtained with inflammatory masses, dermoid cysts and endometriomas while false negative outcomes were noted with early stage cancer or borderline tumours. Later studies showed improved sensitivity (87%) and specificity (100%) in diagnosing a malignant ovarian lesion using amaximum standardized uptake value (SUVmax) of over 3.^5^ This improved performance of ^18^F-FDG PET CT is partly due to its use in collaboration with clinical risk algorithms and improved CT acquisition. ^18^F-FDG PET CT performed in patients with a pelvic mass detected on ultrasound and a risk of malignancy index of greater than 150 (determined by ca125 levels, menopausal status and ultrasound features), provides an improved sensitivity and specificity for diagnosing a malignant lesion of 100% and 92.5%, respectively.^6^ More recently, Dauwen et al prospectively evaluated 69 patients suspected of having ovarian cancer to assess the value of ^18^F-FDG PET CT in staging, diagnosis and surgical resectability. Sensitivity and specificity for the diagnosis of malignancy were 93% and 77%, respectively, compared to 96 and 38% for CT alone. ^18^F-FDG PET CT was both more sensitive and specific compared to CT in the detection retroperitoneal lymph nodes metastases but no significant difference was observed in the detection of peritoneal deposits.^7^

More recent ^18^F-FDG PET CT literature concentrates on the possibility of differentiating between borderline and malignant lesions and between histological subtypes. Kim et al retrospectively reviewed imaging on 13 early FIGO StageI malignant ovarian tumours or borderline tumours and found that SUVmax was significantly lower in the group with borderline tumours.^8^ The SUVmax of the borderline tumours was significantly lower than malignant lesions (2.9 ± 1.5 *vs* 6.6 ± 2.9); however,no significant difference was seen in age at presentation, CA-125, tumour size, or metabolic tumour volume. SUVmax of 3.7 was the best threshold value to differentiate between borderline and malignant tumours with a sensitivity of 83.3% and specificity of 85.7%. Subsequently, Tanizaki et al evaluated 160 patients suspected of having malignant ovarian cancers. In this study, ^18^F-FDG PET CT had a high specificity and PPV of 94.6 and 91.5%, respectively, for differentiating malignant from benign or borderline tumours using SUVmax threshold of 2.9.^9^ When differentiating between subtypes of ovarian cancer, clear cell and mucinous subtypes showed significantly lower FDG uptake compared to serous and endometriod subtypes. Of 14 borderline tumours, only two showed uptake on ^18^F-FDG PET CT. 3.8% were benign false positive lesions (struma ovarii, endometriotic cyst and ovarian abscess) with significant FDG accumulation. Although the performance of ^18^F-FDG PET CT compares favourably against CT, the benign false positives limit its clinical use. No SUVmax values are reliable and reproducible to distinguish between benign, borderline or malignant ovarian masses. To our knowledge, there are no prospective randomized studies comparing the performance of transvaginal ultrasound, MRI and PET CT. Based on the individual study performance data and retrospective studies, ultrasound and MRI remain the primary modes of imaging for the detection and characterization of adnexal masses.

### Staging ovarian cancer

Abdominal and pelvic peritoneal disease is present in more than 70% of females at presentation. The optimal standard of care for patients with ovarian cancer is either primary cyto-reductive surgery or adjuvant platinum based chemotherapy followed by cyto-reductive surgery. The extent and distribution of disease determines whether complete cyto-reduction can be performed with complete resection (R0) or residual disease less than 1 cm (R1) resection offering the patient best chance for cure. The detection of disease at surgically critical sites is essential to ensure appropriate treatment selection for the patient. Imaging plays a pivotal role in determining whether optimal cytoreduction is achievable. Contrast enhanced CT is the most commonly used modality for determining feasibility of optimal surgical cytoreduction. However, the performance of CT varies vastly in the literature, with sensitivities ranging between 7 and 28%. Sub centimetre peritoneal implants are frequently not detectable on CT.^10^ ^18^F-FDG PET CT has been evaluated in several studies to determine whether it improves detection of sub centimetre metastatic lesions or changes patient management.

**Figure 2.**
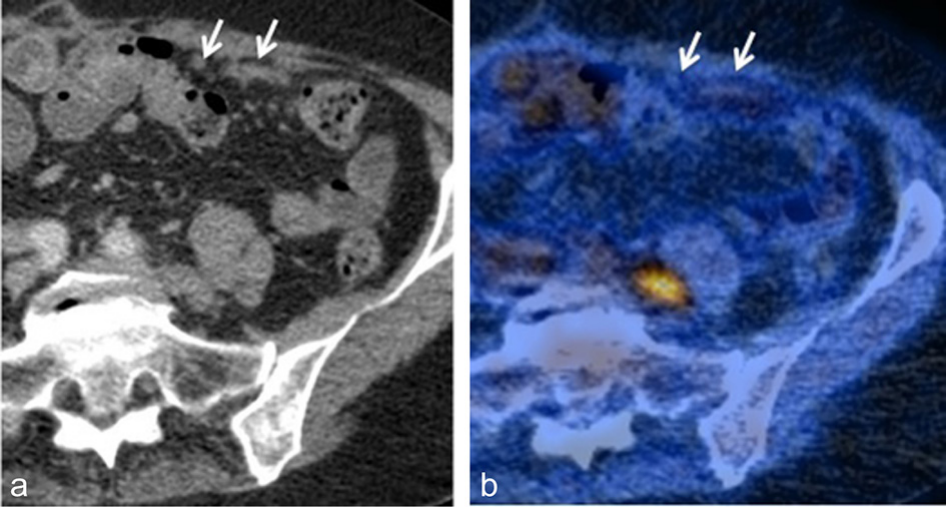
A 72-year-old female with serous carcinoma of the ovary. (a, b) Demonstration of multiple small histo-pathologically confirmed sub centimetre anterior peritoneal nodules not FDG avid but detected on the CT component of ^18^F-FDG PET CT.

In a prospective study encompassing 41 patients, Hynninen et al performed preoperative whole body ^18^F-FDG PET CT followed by diagnostic contrast enhanced CT (CECT) and compared against surgical and pathological findings. Overall, site based analysis showed that ^18^F-FDG PET CT had a sensitivity of 51 *vs* 41% for diagnostic contrast enhanced CT. Specificity for ^18^F-FDG PET CT was 89 *vs* 92% for CT. ^18^F-FDG PET CT had better sensitivity for the detection of large bowel serosal infiltration but this did not hold true for small bowel serosal infiltration. The consensus of this study was that^18^F-FDG PET CT was not unequivocally superior to CE CT alone for the detection of intra-abdominal disease spread^11^ (Figure 2). Other studies similarly show a modest improvement of intra abdominal disease detection, but not significant enough to make a definite clinical impact or alter management and ^18^F-FDG PET CT cannot be reliably used as a stand-alone non-invasive method for prediction of resectability. ^18^F-FDG PET CT failed to detect both sub centimetre and peritoneal deposits > 1 cm in size.^11–13^

Where ^18^F-FDG PET CT performs significantly better than standard diagnostic CECT is in the detection of nodal and distant extra abdominal metastases. Many studies^6,11,14^ have shown that ^18^F-FDG PET CT upstages disease, largely due to identification of supradiaphragmatic metastases not imaged or identified on CECT. In their study, Risum et al showed an increased number of patients with Stage 4 disease when ^18^F-FDG PET CT was used preoperatively in patients with a risk of malignancy index greater than 150. They concluded that the use of this modality as a staging technique would lead to stage migration. Although there is institutional variation, presence of small volume extra abdominal metastases does not alter the decision or approach to surgery for abdominal and pelvic disease. This is still based on the extent, distribution and resectability of abdominal disease. The best patient outcomes still rest on achieving R0/R1 resection of abdominal disease. Consequently, ^18^F-FDG PET CT does not offer an unequivocal benefit over CECT when it detects small volume extra abdominal disease at presentation. In disease less than 1 cm in size, its performance is equivalent to CECT and this remains a challenge.

### Response assessment

There is very limited data on the use of ^18^F-FDG PET CT in the evaluation of treatment response in ovarian cancer. Pre treatment baseline ^18^F-FDG PET CT in patients undergoing chemotherapy serves an important role for post treatment monitoring of disease response to chemotherapy. In a recent study of 26 patients with disease considered unsuitable for primary debulking and treated with neoadjuvant chemotherapy, Vallius et al investigated whether PET CT could identify potential non-responders to neoadjuvant chemotherapy. ^18^F-FDG PET CT findings were compared against diagnostic laparoscopy pre treatment and after 3/4 cycles of chemotherapy. They showed that an omental SUVmax reduction of less than 57% correlated with histopathological non-responders.^15^

A retrospective study conducted by Caobelli et al in 2016 with168 females undergoing restaging PET CT at least 6 months following primary surgery and completion of adjuvant treatment showed that a negative study was associated with a significantly longer progression-free survival and a higher overall survival rate after 4 years of follow up, compared with a positive PET CT at the end of treatment.^16^ Although this shows promise, more data is required to be able to make clinical recommendations for the use of ^18^F-FDG PET CT in disease monitoring and surveillance of ovarian cancer.

### Ovarian cancer recurrence

Up to 75% of patients with ovarian cancer will develop disease recurrence and early identification of the site and extent of recurrence is critical in formulating an appropriate management plan. The tumour marker CA-125 is sensitive in identifying recurrent disease but has a poor specificity and low negative predictive value. Moreover, the total tumour burden cannot be reliably assessed with CA-125. The role of ^18^F-FDG PET CT in the evaluation of ovarian cancer recurrence has been extensively researched.

As early as in 1993, ^18^F-FDG PET CT was found to localize recurrent tumour correlating with surgico-pathological findings. In a small study comprising 13 patients, six patients who were clinically felt to have recurrence, FDG avid disease greater than 1 cm on PET CT showed good concordance with sites of surgico-pathological disease. ^18^F-FDG PET CT had a false negative in five patients who were found to have microscopic disease only on pathological evaluation.^17^

**Figure 3.**
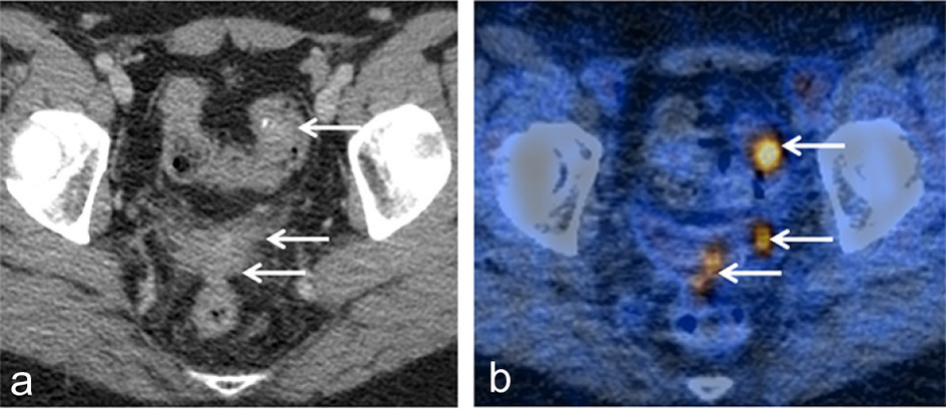
A 78-year-old female with previous serous papillary ovarian carcinoma, 2 years following radical surgery and R0 resection, presents with rising CA 125. (a) Part of diagnostic CT where no unequivocal site of recurrence was seen, but can be identified retrospectively (arrow). (b) Part of the^18^F-FDG PET CT demonstrating laparoscopically confirmed FDG avid disease along the serosal surface of the rectum, sigmoid colon and in the left hysterectomy bed (arrows). FDG, fluoro-2-deoxy-D-glucose; ^18^F-FDG PET CT, [fluorine-18]-fluoro-2-deoxy-D-glucose positron emission tomography. F-FDG PET CT, [fluorine-18]-fluoro-2-deoxy-D-glucose positron emission tomography.

10 years later, several studies reproduce these findings with excellent results in detecting macroscopic disease >1 cm but poor detection of sub centimetre disease. Bristow et al demonstrated in patients with rising biochemical markers and negative or equivocal conventional CT that^18^F-FDG PET CT had a high sensitivity (83.3%) and positive predictive value (93.8%) for detection of macroscopic disease >1 cm.^18^ Simcock et al showed ^18^F-FDG PET CT identified more sites of disease, altering the management for 58% patients with recurrent ovarian cancer. Their study highlighted the contribution of ^18^F-FDG PET CT in the detection of occult disease and for confirming single site disease when surgery is planned^19^ (Figure 3). In a meta-analysis of 34 studies, Gu et al also confirmed the improved sensitivity of detecting disease by PET particularly in patients with a rising CA125.^20^ More recent studies continue to demonstrate limitations of detecting miliary and small <1 cm peritoneal seeding. When compared to CT and MRI, ^18^F-FDG PET CT has greater accuracy in the detection of <2 cm peritoneal implants.^21^ However, this advantage is now challenged by diffusion weighted MRI. Michielsen et al found that whole-body diffusion-weighted MRI showed higher accuracy (91%) in detecting peritoneal disease compared to contrast CT (75%) and ^18^F-FDG PET CT (71%).^21–23^

In recent years, there has been escalating interest in intratumoural FDG heterogeneity. Heterogeneous uptake of the FDG tracer is hypothesized to be due to a combination of factors such as necrosis, angiogenesis and glucose uptake and therefore predictive of disease response to chemo and radiotherapy. Lee et al retrospectively reviewed preoperative ^18^F-FDG PET CT studies of 61 patients who had primary surgery. They analyzed multiple PET indices including maximum SUVmax, total lesion gylcolysis (TLG), Metabolic tumour volume (MTV) and intratumoural FDG heterogeneity (IFH). Of these various PET parameters, high values of IFH were significantly associated with reduced progression-free survival. The authors suggest patients with high IFH could therefore potentially be managed with more intensive chemotherapy regimes.^24^ This new development provides a role for the functional aspect of PET CT in orchestrating optimal patient therapy. However, this exciting prospect still requires further validation prior to clinical application.

### Key points: ^18^ F-FDG PET CT in ovarian cancer

- Has greatest impact for patients with documented or suspected relapse with a rising CA125 considered for salvage therapy.
- at original staging, although it is superior on a lesion by lesion basis compared to contrast enhanced CT alone, this superiority does not translate to significant management change for the patient.
- It is superior to CT alone in characterizing adnexal masses but inferior to the combination of MRI and US.
- It shows promise as a surrogate biomarker to predict chemotherapy response in patients undergoing neoadjuvant chemotherapy

## Role of ^18^F-FDG PET CT in cervical cancer

^18^F-FDG PET CT has a well-established role in the management of patients with cervical cancer. There is good quality evidence in the literature, clear indications and a meaningful uptake of ^18^F-FDG PET CT in clinical practice for the overall management of patients with cervical cancer.

### Detection and staging

Accurate staging at presentation is crucial to plan optimal therapy, and it is one of the most important prognostic factors in patients with cervical cancer. Small tumours confined to the cervix are treated with radical hysterectomy and bilateral salpingo-oophorectomy or trachelectomy in patients wishing to preserve fertility whereas patients with tumours extending into the parametrium or beyond, and/or locoregional nodal disease, will be treated with chemoradiotherapy. In advanced cervical cancer, progression-free survival is significantly related to FIGO stage, patient age, performance status, para aortic and pelvic lymph node metastasis and tumour size.^25^ The latest FIGO staging guidelines have incorporated measurements of tumour size^26^ but not included assessment lymph node metastasis. High-resolution MRI and diffusion-weighted imaging are still the best imaging techniques for initial local staging, evaluating tumour extension into surrounding parametrial tissue and adjacent organs and have shown overall staging accuracy of 80–92%.^27^

Although primary tumours are usually metabolically active, ^18^F-FDG PET CT has a very limited role in the loco-regional primary staging of cervical cancer as the anatomic definition is far superior with MRI. Sugawara et al showed that ^18^F-FDG PET CT detected only 76% of primary tumours despite improving visualization by bladder voiding and dynamic acquisition.^28^

**Figure 4.**
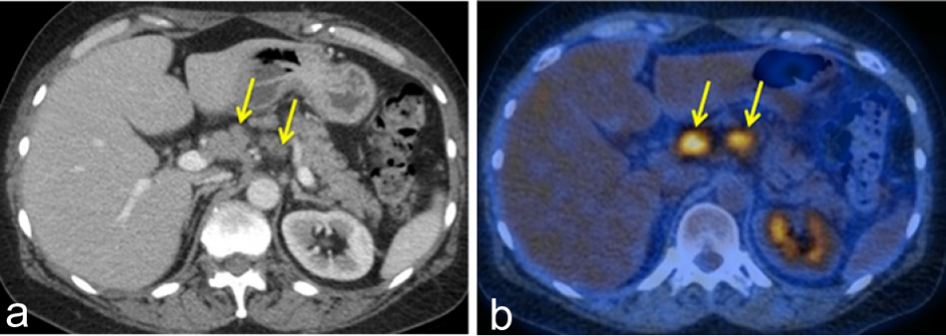
A 35-year-old female with Grade3SCC of the cervix. (a) Demonstration of borderline lymph nodes, suspicious of metastatic disease but at an unusual site in cervical cancer (arrows). The ^18^F-FDG PET CT (b) was performed for further evaluation and demonstrates highly FDG avid lymph nodes, laproscopically confirmed as metastatic SCC lymph nodes (arrows). FDG,fluoro-2-deoxy-D-glucose; ^18^F-FDG PET CT, [fluorine-18]-fluoro-2-deoxy-D-glucose positron emission tomography; SCC, squamous cell carcinoma.

The most important role of ^18^F-FDG PET CT is detection of lymph node and distant metastases where its performance is superior to conventional MRI and contrast enhanced CT (Figure 4). Lymph node status in cervical cancer is one of the most important prognostic indicators, adversely affecting patient prognosis. Many studies have evaluated the role of ^18^F-FDG PET CT in lymph node assessment in cervical cancer and it has become clear that it has an established role in lymph node assessment in advanced cervical cancer. The detection of pelvic and para-aortic nodes influences the extent and delivery of chemoradiotherapy. In early cervical cancer (FIGO Stage IA to IIA) it has a low sensitivity in the detection of nodal metastases, limiting its role. Wright et al performed a retrospective review of 59 patients with early stage cervical carcinoma undergoing ^18^F-FDG PET CT and found that it detected pelvic nodal metastases with a sensitivity of 53%, and a specificity of 90%. Detection of para-aortic nodal metastases had sensitivity of 25%, and specificity of 98% with the mean size of metastastic nodes of 15 mm.^29^ This size dependency is an important factor in the detection of nodal metastases as demonstrated in a prospective study by Sironi et al, including patients with early cervical cancer. ^18^F-FDG PET CT detected metastases in lymph nodes larger than 0.5 cm with a sensitivity of 100% and a specificity of 99.6%, but when nodes of all sizes were included, sensitivity fell to 77%.^30^ Choi et al compared the performance of MRI and ^18^F-FDG PET CT in detection of pelvic and para-aortic nodal metastases in cervical cancer patients with early and advanced disease (Stage Ib–IVa) compared with lympadenectomy of all visible nodes at surgery in 22 patients. ^18^F-FDG PET CT was more sensitive than MRI (58 *vs* 30%), although no significant differences were noted with regard to the specificity (93 *vs* 93%) and accuracy (85 *vs* 73%) of both methods.^31^ A systematic review and meta-analysis including ten studies with 385 patients demonstrated that pooled specificity of FDG-PET CT for detection of para-aortic nodal metastases was consistent at97%, but the sensitivity was low overall at34%. This sensitivity was heterogeneous and higher in studies with a higher prevalence of nodal disease.^32^ Consequently, although the sensitivity of detecting small nodal metastases is poor for all modalities, ^18^F-FDG PET CT offers the best sensitivity in advanced cervical cancer.

#### Treatment response and prediction of survival outcome

^18^F-FDG PET CT has been shown by several authors to reliably assess treatment response and predict survival outcome. Persistent FDG uptake at the end of treatment with chemo radiotherapy confers a poor 5-year survival outcome. Grigsby et al retrospectively evaluated ^18^F-FDG PET CT imaging from 152 patients who underwent both pre and post treatment scans.^33^ In the 114 patients with no residual FDG uptake on the post treatment scans, the 5-year survival rate was 92%. For patients with persistent or new FDG uptake, 5-year survival rate was 46 and 0%, respectively. Schwarz et al then validated these findings in a prospective study with 92 patients imaged at an average of 3 months following end of treatment. On ^18^F-FDG PET CT, 65 patients had a complete response, 15 had a partial metabolic response and 12 had disease progression.^34^ 3-year progression-free survival rates were 78, 33 and 0%, respectively. Interestingly, the combination of nodal metastases detected and disease response as assessed on ^18^F-FDG PET CT, predicted progression-free survival, while clinical stage did not. Kidd et al who prospectively studied 287 patients with Stage Ib to IVb cervical cancer have also evaluated the potential role of SUVmax of the primary cervical tumour and the mean SUV of metastatic pelvic nodes as a biomarker for survival parameters. They showed no correlation between tumour volume and SUVmax, between squamous and non-squamous histology but higher SUVmax was associated with an increased risk of lymph node metastasis at diagnosis. The overall survival rates at 5 years were 95% for an SUVmax ≤ 5.2, 70% for an SUVmax >5.2 and ≤ 13.3, and 44% for an SUVmax >13.3. Higher SUVmax values correlated with a higher risk of persistent FDG uptake at the end of treatment. The SUV of metastatic nodes can also provide prognostic information. Higher values of the mean SUV of pelvic metastatic nodes correlates with an increased risk of persistent disease at the end of treatment and is an independent predictor of developing pelvic disease recurrence.^35^ The mean SUV of pelvic nodal metastases show no significant correlation with the nodal size, primary cervical tumour size or SUVmax, or the mean SUV of para-aortic nodal metastasis.

The precise time points for the routine use of ^18^F-FDG PET CT in asymptomatic patients for surveillance and recurrence is summarized in Table 1. The Scottish Intercollegiate Guidelines Network (SIGN) 2008, recommend a ^18^F-FDG PET CT 9 months following completion of chemotherapy while the American National Comprehensive Cancer Network (NCCN) guidelines recommend ^18^F-FDG PET CT 3–6 months after completion of treatment. In symptomatic suspected recurrence, the RCR, SIGN and NCCN guidelines recommend ^18^F-FDG PET CT when cross-sectional imaging is equivocal or patients are being considered for salvage surgery.^36–39^ The key role of FDG PET-CT is early detection loco-regional treatment failure suitable for salvage therapy and to identify unsuspected asymptomatic distant metastatic disease. The performance of ^18^F-FDG PET CT in detecting residual disease has been evaluated in several small studies, with the most recent study by Scarsbrook et al, demonstrating sensitivity and specificity of 94.3 and 62.3%, respectively. The positive predictive value was 58.9% and the negative predictive value was 95.0% with an overall diagnostic accuracy of 74%. All patients with residual nodal uptake greater than background liver activity went on to relapse in this study. The ^18^F-FDG PET CT was performed 8–16 weeks after completion of chemo radiotherapy. The false positive FDG uptake, lowering the specificity and positive predictive value, was secondary to radiotherapy induced inflammatory changes.^40^ In a large study by Schwarz et al, evaluating ^18^F-FDG PET CT response patterns and relapse, all patients with persistent disease at completion of treatment had disease relapse, 65% with partial response and 23% of patients with complete metabolic response on ^18^F-FDG PET CT had loco-regional or distant metastases within 18 months.^41^ In this study, all patients had received a combination of external beam radiotherapy and intracavitary brachytherapy and the majority (92%) had received chemotherapy. In recent years, there has been increasing interest in the use of MRI and ^18^F-FDG PET CT to improve tumour delineation for radiotherapy planning in cervical cancer. Detection and inclusion of lymph node metastases in the pelvis and para-aortic distribution for radiotherapy treatment volume is the single most important improvement ^18^F-FDG PET CT can offer over cross-sectional imaging alone. Inclusion of the nodes lowers local recurrence but has little impact on overall survival.^42^ False negative results occur in 12% of para-aortic nodes,^43^ and despite lymphadenectomy these patients have a poor survival rate and the benefit of treating non-FDG avid occult metastases remains unclear.

^18^F-FDG PET CT can also be integrated in radiotherapy treatment planning for dose escalation and simultaneous boost regimes to achieve increased tumour shrinkage and likelihood of local control.^44^ The challenge however remains, as most patients relapse with distant disease, but the integration of functional MRI techniques or ^18^F-FDG PET CT in delivering radiotherapy improves local disease control and quality of life for the patient.

### Recurrent disease

**Figure 5.**
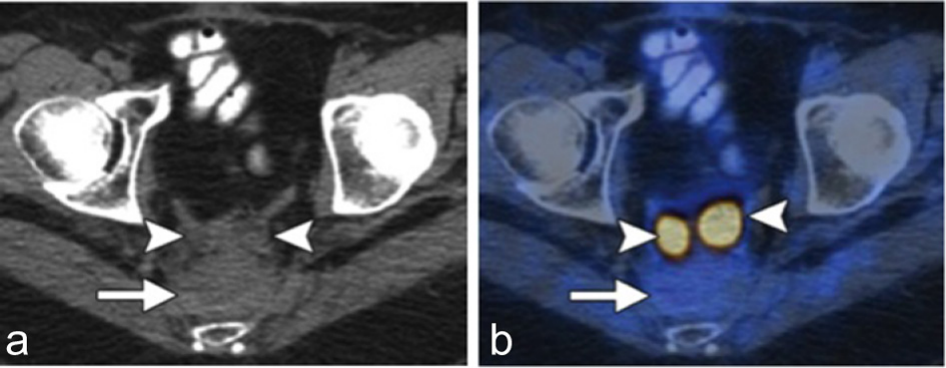
26-year-old female with Grade 2 SCC of the cervix, following radical TAH BSO and EBRT. Surveillance CT (a) demonstrates that non-specific changes in the pre-sacral space (arrow) and hysterectomy bed (arrow heads) and recurrence would be indistinguishable from radiotherapy changes. (b) Demonstration of FDG avid biopsy confirmed recurrent disease in the hysterectomy bed (arrow) and no recurrence in the pre-sacral space (arrow heads). EBRT, external beam radiotherapy; FDG, fluoro-2-deoxy-D-glucose; SCC, squamous cell carcinoma.

Recurrence is defined as development of tumour at least 6 months after the treated disease has regressed. The detection and precise localization of recurrent disease is critical, guides management and selection of appropriate therapies, prolonging survival. A few retrospective studies have evaluated the role of ^18^F-FDG PET CT in recurrent cervical carcinoma. In a study by Kitajima et al with 52 females who had completed treatment for cervical cancer and had suspected recurrence, ^18^F-FDG PET CT had a sensitivity of 92% with a specificity of 92.6%.^45^ A more recent systematic review and meta-analysis of eight studies with 221 patients, found sensitivity of 94% and a specificity of 84% on per patient basis for detection of recurrence^46^ (Figure 5). In most of the studies, there remain false negatives on ^18^F-FDG PET CT in small non-FDG avid local recurrences and small sub centimetre para aortic nodes. False positives occur due to benign mediastinal nodes, well-known pitfalls of ^18^F-FDG PET CT. In addition, imaging patients too soon after radiation treatment may lead to false positive FDG uptake secondary to post radiotherapy inflammatory change.

**Figure 6.**
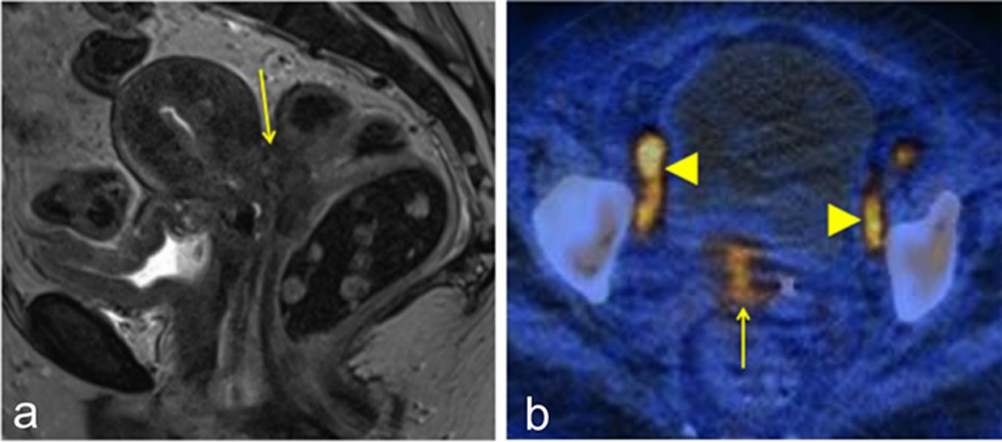
A 32-year-old female with SCC of the cervix, FIGO Stage IIb, following EBRT. (a) Sagittal T2-weighted image of the pelvis, demonstrates residual disease in the cervix, pouch of Douglas and rectal serosa (arrow). The MRI appearances suggested suitability for pelvic exenteration, and ^18^F-FDG PET CT was performed for confirmation. (b) ^18^F-FDG PET CT demonstrates FDG avid bilateral subcentimetre external iliac nodes excluding exenteration. The right-sided FDG avid node was biopsied and confirmed metastatic SCC. EBRT,external beam radiotherapy; FDG, fluoro-2-deoxy-D-glucose; ^18^F-FDG PET CT, [fluorine-18]-fluoro-2-deoxy-D-glucose positron emission tomography; SCC, squamous cell carcinoma.

The high accuracy in the detection of recurrent disease makes^18^F-FDG PET CT suitable for selecting patients considered suitable for exenterative surgery (Figure 6). The exclusion of distant disease and confirmation of a small central recurrence is the prime objective. This surgery carries a high morbidity and mortality, and identification of extra pelvic disease would render a patient unsuitable for this salvage procedure. Burger et al in a retrospective study of 33 patients (18 with cervical cancer) demonstrated that ^18^F-FDG PET CT was able to accurately select suitable patients and the quantitative metrics of FDG uptake incorporating MTV can serve as predictive biomarkers of progression-free and overall survival.^47^

### Overall, in cervical cancer, ^18^ F-FDG PET CT

- Improves detection of distant and nodal metastatic disease in advanced cervical cancer, making it the modality of choice for staging. Its sensitivity is just under 80%, and although it is the most accurate imaging modality availability, 18F-FDG PET CT does not match the performance of laparoscopic nodal dissection.
- Performs functional evaluation of the SUVmax of the primary tumour and mean SUV of pelvic nodes provides functional evaluation that can be used as a biomarker for response, survival parameters and predicting disease recurrence.
- Has an established role in detecting distant recurrent disease and is therefore essential when selecting patients for exenterative surgery.

## Role of ^18^F-FDG PET CT in endometrial cancer

### Detection and staging

Endometrial cancer is now the most common gynaecological cancer in the Western world. The role of ^18^F-FDG PET CT in endometrial cancer has evolved over the past decade with a wider range of applications in the care of patients with endometrial cancer. ^18^F-FDG PET CT is emerging as a useful adjunct to conventional imaging in the detection of unexpected nodal or distant disease in young patients being considered for pelvic exenterative surgery for central recurrence of endometrial cancer.

^18^F-FDG PET CT has no established role in the detection and local staging of endometrial cancer. A meta-analysis looking at the performance of ^18^F-FDG PET CT for the detection of endometrial cancer in a high risk group showed a moderate sensitivity (81.9%) and specificity (89.8%) for primary endometrial cancer. The performance of ^18^F-FDG PET CT is limited in detecting the primary tumour as there is normal physiological endometrial FDG uptake in pre and peri-menopausal females during the normal ovulatory and menstrual phases of the menstrual cycle. For similar reasons and also due to the lack of spatial resolution in defining the tumour, ^18^F-FDG PET CT has limited value in the evaluation of depth of myometrial invasion.^48^

The risk of nodal metastases, which negatively impacts on patient survival, is dependent on the depth of myometrial invasion, cervical tumour involvement, adverse tumour subtype and histological lympho-vascular invasion. The overall risk of lymph node metastases is less than 10% in Stage I disease, except in Grade 3 tumours when the risk is 18% and there is a three-fold greater risk of lymph node metastases in Stage II disease (cervical invasion).^49^ The depth of myometrial invasion, however, is probably the single most important prognostic factor and in Grade3 tumours, there is a 9% incidence of nodal metastases with superficial invasion (<50%), but this increases to 34% when deep myometrial invasion (>50%) was present. In patients with no myometrial invasion and low-grade histology, the 5-year survival rate is 95%, compared to 42% in patients with tumour invasion extending into the outer half of the myometrium and high-grade histology.^49–51^

**Figure 7.**
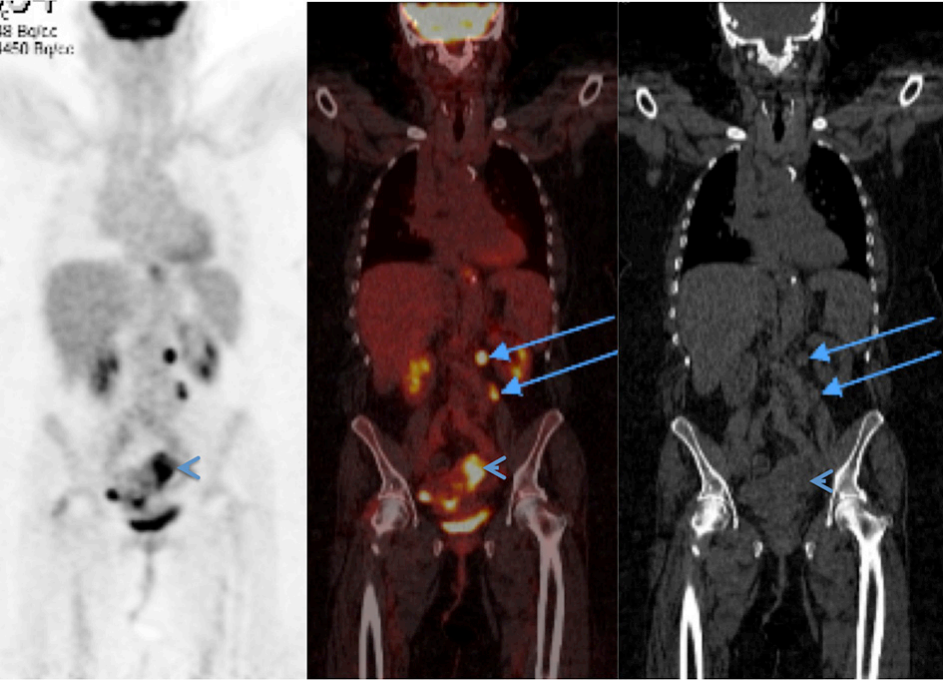
A 68-year-old female with Grade 3 endometriod endometrial carcinoma. Staging CT demonstrated suspicious left para-aortic nodes (arrows). A PET CT was performed demonstrating avid FDG tracer uptake in the nodes confirming metastases (arrows), in keeping with FIGO Stage IIIC2 endometrial carcinoma. The open arrow highlights the endometrial carcinoma, which also has avid FDG uptake. FDG, fluoro-2-deoxy-D-glucose; PET CT, positron emission tomography.

Patients with deep myometrial invasion have a six to seven-fold increase in the prevalence of para-aortic or pelvic lymph node metastases compared to patients with less than 50% invasion (Figure 7) . Although lymphadenectomy is part of FIGO staging, its role, extent (pelvic *vs* combined pelvic and para-aortic; sampling versus complete dissection; systematic dissection *vs* selective dissection) and therapeutic effect are an issue of great debate. There is also conflicting evidence in the literature regarding the therapeutic benefits of lymphadenectomy and it therefore becomes debatable whether detection of nodal micro metastases is clinically relevant.

A large randomized controlled trial with 514 patients showed that 5-year disease-free and overall survival rates were very similar in patients undergoing pelvic lymphadenectomy (81.0 and 85.9%) and in patients not undergoing lymphadenectomy (81.7 and 90.0%).^52^ Both early and late postoperative complications occurred statistically significantly more frequently in patients who had received pelvic lymphadenectomy. Similarly, the ASTEC study group with 1408 patients also concluded that there was no evidence of benefit in terms of overall or recurrence-free survival for pelvic lymphadenectomy in females with early endometrial cancer.^53^ In the ASTEC trial, the follow-up period was short and lymphadenectomy was selective rather than systematic. Nine or fewer lymph nodes were removed in 35% of patients, despite the fact that removal of at least ten pelvic nodes has been shown to be needed for a survival benefit. Secondly, no para-aortic lymphadenectomy was performed, negating the therapeutic effect of lymphadenectomy as > 50% of patients with pelvic lymph node metastasis have para-aortic metastasis, and about 10% of lymph node metastases skip the pelvis and occur exclusively in the para-aortic region. The SEPAL study, from Japan, has challenged the findings of ASTEC and shown a significant improvement in overall, disease-specific and recurrence-free survival in patients with intermediate or high risk disease (not in low risk patients) who undergo complete pelvic and para-aortic nodal dissection compared with pelvic dissection alone.^54^

Only 20–25% of high-risk patients will present with nodal metastases and therefore routine systematic lymphadenectomy in all patients is not recommended. Although practice varies greatly in the use of lymphadenectomy in patients with high and moderate-risk endometrial cancer, radical surgery with lymphadenectomy is generally reserved only for high-risk patients. A non-invasive technique accurately identifying lymph node metastases would obviate the need for routine staging lymphadenectomy in all patients, reducing morbidity and allowing appropriate selection of patients. Crivellaro et al evaluated the role of ^18^F-FDG PET CT in preoperative nodal staging of high-risk clinical StageI endometrial cancer. High-risk patients were defined as having Grade2 tumours with deep myometrial invasion, all Grade3 tumours or any grade serous/clear cell pathology. ^18^F-FDG PET CT had a moderate sensitivity of 78.6% and a high specificity of 98.4% for lymph node metastases. The recent ACRIN study enrolling 215 patients sensitivities of ^18^F-FDG PET CT versus diagnostic CT for the detection of lymph node metastasis were 65 *vs* 50% in the abdomen and 65 *vs* 48% in the pelvis. Specificities were 88 *vs* 93% in the abdomen and 93 *vs* 89% in the pelvis.^55,56^ When nodal size is considered, Kitajima et al showed 17% sensitivity for nodes ≤ 4 mm, 67% sensitivity for nodes between 5 and 9 mm and 93% sensitivity for nodes ≥ 10 mm for ^18^F-FDG PET CT despite the integration of CECT with PET.^45^ Overall, the sensitivities and specificities for lymph node staging were 72.3 and 92.9% and for distant metastasis detection were 95.7 and 95.4%, respectively. These improved overall sensitivity figures are largely influenced by the improved sensitivity of ^18^F-FDG PET CT with increasing nodal size. From the literature, unfortunately ^18^F-FDG PET CT continues to provide only modest improvement in the detection of microscopic nodal metastasis in patients with high-risk endometrial cancer and therefore cannot be justified routinely for the selection of patients considered for lymphadenectomy.

### Endometrial cancer recurrence

A recent systematic review and meta-analysis by Bollineni et al focusing on lymph node staging and recurrence in endometrial cancer after primary surgical treatment included eight studies on endometrial cancer recurrence. They showed ^18^F-FDG PET CT had pooled sensitivities and specificities of 95 and 91% in the detection of recurrent endometrial cancer.^57^ The largest retrospective study included in this meta-analysis included 127 patients who showed no evidence of residual disease following initial therapy for endometrial cancer. 15% of patients had a recurrence detected by ^18^F-FDG PET CT but not by conventional imaging methods. In a meta-analysis of 541 patients by Kadkhodayan et al the sensitivity and specificity for the detection of recurrent disease was 95.8 and 92.5%, respectively. Although this result is very favourable, the meta-analysis is only based on retrospective single centre studies with great heterogeneity and no mandatory histopathological verification.^58^ Post surgical changes and fibrosis often hamper the detection of early or small volume recurrence on conventional MRI or routine contrast enhanced CT. ^18^F-FDG PET CT also excludes the presence of distant metastases more reliably than contrast enhanced CT. As for ovarian and cervical cancer, patients being considered for exenterative surgery for pelvic recurrence of endometrial cancer would benefit from preoperative ^18^F-FDG PET CT to exclude distant unsuspected visceral and nodal metastases.

### ^18^F-FDG PET CT as predictor of outcome and biomaker

For endometrial cancer, multiple studies have demonstrated that increasing tumour size correlates with increasing metastatic nodal potential and poor prognosis.^59,60^ On ^18^F-FDG PET CT, the increasing metabolic activity of the primary tumours is also linked to poor prognosis. Antonsen et al in their study of 268 patients, demonstrated that SUVmax was significantly higher in patients with high FIGO stage and deep myometrial invasion.^61^ Lee et al in there study showed increasing SUVmax with FIGO stage, histologic grade, histological lympho-vascular space invasion, and maximum tumour size. Walentowicz-Sadlecka et al revealed that SUVmax threshold value of > 17.7 predicted high risk of recurrence and lower overall survival.^62,63^ High SUVmax, mean SUV, MTV, and TLG are predictors of deep myometrial invasion, cervical stroma invasion, lymph node metastases, and poor prognosis in endometrial cancer. The thresholds for these parameters in identifying high-risk patients have a wide range in the literature: for SUVmax > 9–18, for MTV > 9–30 ml and for TLG > 56–70 g.^61,63^ This variation in thresholds reflects the lack of standardization of imaging protocols and post-processing methods within studies. Further studies or expert guidelines are needed to validate and standardize metabolic ^18^F-FDG PET CT parameters to optimize thresholds allowing risk stratification for potential clinical use.

Recent studies have evaluated the degree of FDG uptake in the primary tumour prior to treatment and its association with survival outcomes. High SUVmax and TLG corresponds to tissue high hypoxia markers such as stromal HIF-1α in endometrial cancer, which confers a significantly reduced disease specific survival.^64^

### Overall for endometrial cancer

- Overall for endometrial cancerThe main established role for ^18^F-FDG PET CT in endometrial cancer is for the detection of extra uterine disease. Reported accuracies for preoperative nodal staging of endometrial cancer by ^18^F-FDG PET CT modestly better than conventional imaging are nodal size dependent and still not good enough to replace lymphadenectomy.
- ^18^F-FDG PET CT is excellent in the detection of recurrent disease, and in patients considered for exenterative surgery, it is recommended to exclude unsuspected distant metastases.
- The potential added value of ^18^F-FDG PET CT as a predictive biomarker is promising but requires improved standardization and validation of metabolic threshold values.

### ^18^F-FDG PET-MRI

Combined ^18^F-FDG PET and MRI (PET MRI) is unique clinical imaging modality offering significant potential advantages for patients including reduced radiation dose, better anatomical information offered by MRI over CT, which may prove to be an essential tool to deliver personalized gynaecological cancer care. Despite some technical challenges, such as correcting for photon attenuation, PET MR is emerging as an important clinical and investigative tool. For gynaecological cancers, the main advantage is in the complete assessment of locoregional tumour extent and whole body evaluation for nodal, peritoneal and skeletal metastases. It has been proposed for staging, mapping gross tumour volume for delivery of radiation therapy in cervical cancer, assessing chemotherapy response in endometrial and ovarian cancers and detecting suspected recurrent disease in most gynaecological cancers. The preliminary studies including all gynaecological malignancies show that ^18^F-FDG PET MRI is superior to ^18^F-FDG PET CT for primary tumour delineation and may eventually be the preferred imaging modality for staging cervical and endometrial tumours. Although, it is a reliable tool in the detection of extra abdominal distant metastasis, it does not confer any significant advantage over ^18^F-FDG PET CT in the detection of nodal or distant metastases.^65,66^

## Conclusion

^18^F-FDG PET CT has assumed increasing importance in the management of patients with gynaecological cancers in recent years. This trend reflects a desire to obtain accurate staging, early disease detection, to influence optimal treatment selection and minimize morbidity and mortality. Our review focused on highlighting the strengths and weaknesses of ^18^F-FDG PET CT by interrogating the available literature, thereby guiding the optimal clinical application. Future assimilation and quantitative assessment of both the morphological and functional biological data will provide added value as early indicators of treatment response.
